# Multiple Sclerosis: A Story of the Interaction Between Gut Microbiome and Components of the Immune System

**DOI:** 10.1007/s12035-025-04728-5

**Published:** 2025-02-11

**Authors:** Esraa Mohsen, Hesham Haffez, Sandra Ahmed, Selwan Hamed, Taghrid S. El-Mahdy

**Affiliations:** 1 Department of Microbiology and Immunology, Faculty of Pharmacy, Helwan University, PO Box 11795, Cairo, Egypt; 2 Department of Biochemistry and Molecular Biology, Faculty of Pharmacy, Helwan University, PO Box 11795, Cairo, Egypt; 3 Center of Scientific Excellence “Helwan Structural Biology Research (HSBR), Helwan University, Cairo, 11795 Egypt; 4 Department of Neurology, Faculty of Medicine, Cairo University, Cairo, Egypt; 5 Department of Microbiology and Immunology, Faculty of Pharmacy, Modern University for Technology and Information (MTI), Cairo, Egypt

**Keywords:** Multiple sclerosis, Immunity, B cells, T cells, Gut microbiota, Gut metabolites

## Abstract

Multiple sclerosis (MS) is defined as an inflammatory disorder that chronically affects the central nervous system of young people mostly and is distributed globally. It is associated with degeneration and demyelination of the myelin sheath around the nerves, resulting in multiple neurological disability symptoms ranging from mild to severe cases that end with paralysis sometimes. MS is one of the rising diseases globally that is unfortunately associated with reduced quality of life and adding national economic burdens. The definite MS mechanism is not clearly defined; however, all the previous researches confirm the role of the immune system as the master contributor in the pathogenesis. Innate and adaptive immune cells are activated peripherally then attracted toward the central nervous system (CNS) due to the breakdown of the blood–brain barrier. Recently, the gut-brain axis was shown to depend on gut metabolites that are produced by different microorganisms in the colon. The difference in microbiota composition between individuals is responsible for diversity in secreted metabolites that affect immune responses locally in the gut or systemically when reach blood circulation to the brain. It may enhance or suppress immune responses in the central nervous system (CNS) (repeated short forms); consequently, it may exacerbate or ameliorate MS symptoms. Recent data showed that some metabolites can be used as adjuvant therapy in MS and other inflammatory diseases. This review sheds light on the nature of MS and the possible interaction between gut microbiota and immune system regulation through the gut-brain axis, hence contributing to MS pathogenesis.

**Supplementary Information:**

The online version contains supplementary material available at 10.1007/s12035-025-04728-5.

## Introduction and Background

Multiple sclerosis (MS) is a chronic neurodegenerative disease that is characterized by inflammation, demyelination of the protective layer (myelin sheath) surrounding nerve cells [1]. The most affected parts are the periventricular region, optic nerve, brainstem, spinal cord, proximal cortex, and cerebellum [2]. The affected CNS parts lead to weakness in extremities, sensation abnormalities, vision disorders, dyssynergia, and psychiatric disorders in addition to other neuronal complications [3]. Approximately 2.8 million persons between the age of 20 and 40 years all over the world were diagnosed with MS; however, a pediatric-onset MS was reported during childhood [4].

Recent researches highlight the correlation between integrity of gut microbiota, microorganisms that colonize the gastrointestinal tract (GIT), and host functions, including metabolism, immunity, nutritional responses, circadian rhythmicity [5], as well as central nervous system (CNS) function in diseased and healthy people. Microbiota composition differs from person to person due to multiple factors such as location, sex, age, diet, race, therapies (particularly antibiotics), smoking, gastrointestinal infections, stress, and other individual factors [6, 7].

Nature of MS, role of different cytokines in its pathogenesis, and impact of microbiota on CNS disorders are discussed in previous review articles [8]. In the current review article, we discussed deeply the pathogenesis of MS and role of immune cells, and eventually correlate the impact of gut microbiota and its metabolites with MS onset and prognosis.

## Nature and Etiology of MS

Till now, the onset of MS and the full pathological pathways are not fully understood [1]; there is a debate whether MS is immune-mediated or autoimmune disease due to the absence of the primary criteria of autoimmune diseases which is the presence of a specific auto-antigen [9]. Autoimmune disease occurs when adaptive immunity is directed against self-antigens, causing the body to mistakenly attack normal cells [10]. However, MS is classified as an organ-specific disease (infects the brain and spinal cord specifically) with immune-mediated myelin damaging etiology, but it lacks specific autoantigen. Indeed, till now there is no proven implication of an infectious agent as a cause of immune cross-reactivity in MS patients [9].

The autoimmune reaction against myelin as a full etiological explanation for MS has been challenged due to a number of observations [11]. **Firstly**, some studies showed that the early events of MS are characterized with oligodendrocytes and myelin loss, as well as T cells and B cells absence, implying that MS pathology may depend on a process “other than cell-mediated immunity” [12, 13]. **Secondly**, the presence of large myelin loss lesions in pyramidal and sensorial pathways, mostly caused by infiltrating immune cells [14, 15]. **Thirdly**, demyelinating lesions seen in MS patients after autologous bone marrow transplantation have few or no T cells, suggesting that an intrinsic pathological process in the CNS is the cause of these lesions [16].

Finally, the inside out theory, recent studies highlighted the involvement of peripheral T and B cells that pass through impaired blood–brain barrier to CNS attacking the myelin sheath. On the contrary, inside the CNS accumulation of biochemical changes due to metabolic dysfunction in the CNS leads to demyelination that triggers release of inflammatory mediators [17, 18].

**Collectively**, there are heterogeneous inflammatory and immune episodes that happen in MS, but the relation between MS and antibodies against the myelin oligodendrocyte glycoprotein (MOG) as well as myelin or non-myelin directed autoantibodies has been intensively studied but with inconsistent results [19, 20].

### The Clinical Course of MS

**Fig. 1:**
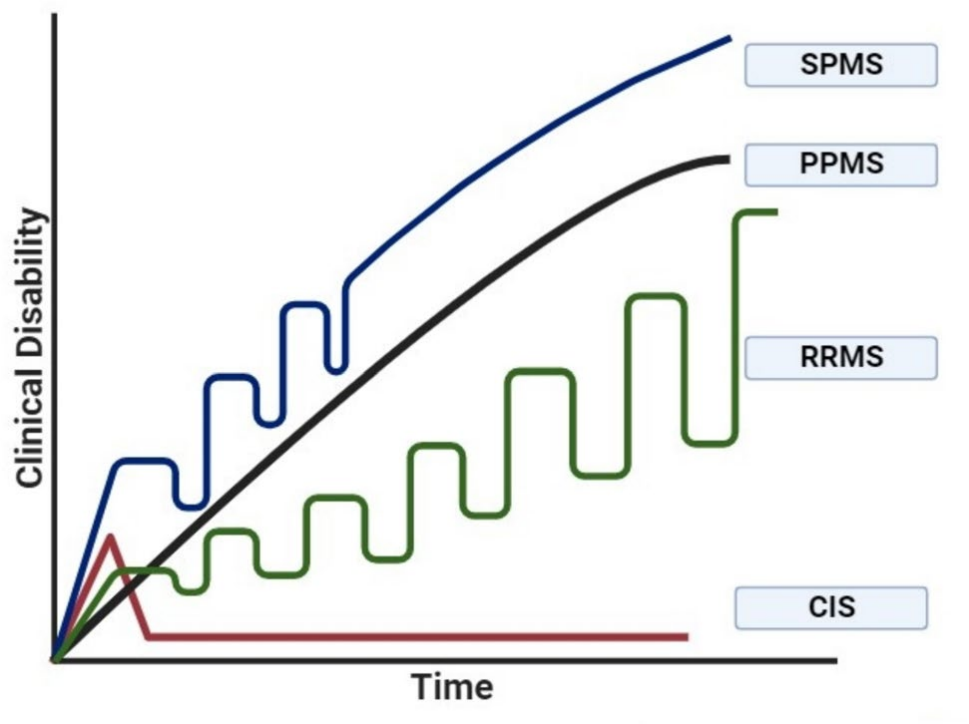
The clinical categories of MS onset; secondary progressive MS (SPMS), primary progressive MS (PPMS), relapsing–remitting MS (RRMS), and clinically isolated syndrome (CIS)

The clinical course of MS is categorized into four categories: (**1**) Clinically isolated syndrome (CIS); the first neurologic symptoms occur due to neuronal inflammation and demyelination in CNS that must extend to 24 h or more, and it may or may not develop to MS [21]. (**2**) Relapsing–remitting MS (RRMS); the most prevalent category, in which new or increased neurological attacks (relapses) happen [21]. These attacks are followed by partial or complete recovery periods (remission). (**3**) Secondary progressive MS (SPMS); this category is characterized by the development of permanent neurological deterioration with prominent clinical disability progression disease form followed by a phase of relapses and remissions [22]. (**4**) Primary progressive MS (PPMS); this category is characterized by increasing disease progression from the start of the disease course [21] (Fig. 1).

### MS Immunopathogenesis: Interaction Between Innate and Adaptive Immunity

MS is considered a result of interaction between adaptive and innate immunity in the periphery through myeloid cells, B cells, T cells, and the CNS through resident cells such as microglial cells and astrocytes [23]. The later cells secrete diverse neurotoxic inflammatory molecules (as reactive oxygen species (ROS), many chemokines and cytokines) that induce the recruitment of different inflammatory cells toward the CNS [24]. Different immune cells released cytokine and their impact on MS are listed in (S1).

#### Role of Adaptive Immunity Role in MS

**Impact of T cells.** The activated T cells (CD4^+^‏ and CD8^+^ T cells) can pass the BBB, initiating a series of reactions through cytokines secretion to exert their effector functions and recruit additional immune cells to start the inflammatory process and formation of an inflammatory demyelinating lesion [25]. T cells are classified into two main subsets, CD4^+^ helper T cells and CD8^+^ cytotoxic T cells; CD4^+^ helper T cells identified subsets are Th1, Th2 cells, Th17, Th22, the regulatory type 1 cells (Tr1), induced T-regulatory cells (iTreg), T helper 9 (Th9), and follicular helper T cell (Tfh) [26, 27]. Several studies, on experimental autoimmune encephalitis model (EAE) [28], have proven the central role of CD4^+^‏ T lymphocytes in MS initiation and development; mainly CD4‏^+^ T helper 1 (Th1), T helper 17 (Th17) cells [29], and CD4^+^ Th2 cells are implicated in MS pathogenesis [30].

Activated T cells, Th1 and Th17 subsets, secrete pro-inflammatory cytokines such as interferon (IFN)-γ, tumor necrosis factor (TNF)-α, Granulocyte–Macrophage Colony-Stimulating Factor (GM-CSF), and IL-17, leading to inflammation, demyelination, and axonal damage [31]. Th1 cells activate macrophages and microglia, while interfering oligodendrocytes survival and differentiation [32]. Th17 cells have a role in disrupting the BBB that facilitates inflammatory immune cells infiltration, also interfere with glial cells functions, and stimulate axonal damage and neurodegeneration [33]. Pathogenic IFN-γ + IL-17 + TH17 cells have been shown to be increased in MS relapses, and increased IL-10 + IL-17 + TH17 cells in stable RRMS patients [34]. IL-23 promotes the conversion of intestinal non-pathogenic TH17 cells into pathogenic CXCR6 + TH17 cells in EAE [35, 36] and observed that GM-CSF produced by pathogenic T cells in MS and EAE promotes differentiation of Ly6Chi monocytes into inflammatory macrophages in presence of IFN-γ [32], and stimulate disease-promoting astrocyte subsets differentiation [33].

Regarding CD8 + T cells, it is considered the predominant T cell population in MS lesions [33].

CD8^+^ T cells cause axonal dissection by releasing cytolytic granules that aggravate the demyelination process [37]. IFN-γ- and IL-17 secreted by activated T cells can activate resident immune cells of the CNS (as astrocytes and microglial cells) [38]. Besides, CD8^+^ T cells also can induce cytokine-mediated death of both endogenous myelin-producing cells [39] and oligodendrocytes leading to neuronal damage once reach the CNS [40]. This activates antigen-presenting cells (APCs), releasing reactive nitrogen species (RNS)and ROS as well as increasing the production of more cytokines [41]. Some CD8^+^ T cells in response to a specific antigen exhibit oligoclonal expansion; these oligoclonal expanded cells were shown to be present in the CSF and CNS, as well as the blood of a small number of investigated patients with MS [42].

Recently, tissue-resident CD8 + T cells were identified as putative drivers of compartmentalized autoimmune CNS damage [43]; recent studies have re-invigorated the study of CD8 + T cells in EAE [44].

**Regulatory T cells** In contrast to the harmful effect of the inflammatory T cell subsets, there are other T cell subsets that can regulate immune activation reactions and control the development of autoimmunity; regulatory T cells (Treg) cells have a protective role in MS [45]. In MS, two subsets of CD4^+^ Treg cells have been identified and investigated. CD4^+^ FoxP3^+^ Tregs are one of the Treg cells subsets that have the capability to decrease the proliferation of T cells in vitro through the expression of the Forkhead box protein 3 (FoxP3) (transcription factor), in addition to a variety of inhibitory surface molecules [46].

Tr1 cells are another type of CD4^+^ Treg cell with proliferation inhibition ability through IL-10 secretion [47]. For example, the transfer of Treg cells specific for myelin oligodendrocyte glycoprotein provided protection against EAE that is achieved in a dose-dependent way [48]. Many studies demonstrate that inflammation in MS may be a result of Treg cell number reduction [49, 50], or functionality [51, 52].

**Role of B cells** Various studies have proven that B cells’ role in MS is not antibody dependent, but rather depends on cellular immunological interactions in the periphery with their capacity to recruit and activate myeloid cells and T cells in the CNS (Li et al., 2018b). These effects were caused by a change in the cytokine secretion pattern, an increase in pro-inflammatory cytokines such as IL-6 [53], TNF-α, and lymphotoxin-α (LT), and production of inflammatory cytokines IL-35 and IL-10 [23] and granulocyte–macrophage colony-stimulating factor (GM-CSF) [54] and aid in the development of Th1 and Th17 cells [54]. Besides a decrease in the regulatory cytokines like IL-10, IL-35, and transforming growth factor-β (TGFβ) and presentation of antigen to T cells IL-21 secreted by pathogenic Th17 cells might promote memory B cell survival and proliferation [55]. Therefore, memory B cells in MS have the ability to induce the activation of T cells and exhibit enhanced pro-inflammatory properties [55]. Mature naive B cells in MS showed abnormal proliferation and activation [56].

#### Implication of B cell Populations in MS

The B cells implication in MS relapses development has discovered after the remarkable outcomes of using selective B cell-–targeting treatments (such as anti-​CD20 antibodies) in MS; however, 95% of MS patients have an abnormal increase of CSF-​restricted IgG oligoclonal bands (OCBs) within the CNS that generated from clonally expanded Ig-secreting cells that does not express CD20 [57]. The success in relapses decreases after anti-​CD20 antibodies therapies were not associated with reduction of CSF Ig profile in MS patients [58], that may indicate that antibodies are not the sole contributor in disease pathology and the role of B cells in MS relapses is not antibody dependent [59]. Indeed, some researchers showed that antibodies can be used diagnostically for some time but are not pathognomonic in MS [59, 60]. Conversely, Roshan et al. believed that antibody-dependent mechanisms may influence disease pathology [61]; in addition, it was shown that IgG bound specifically to myelinating oligo dendrocytes [62], and the complement-dependent demyelination by IgG was found in around 30% of MS patients [63].

The role of B cells in MS may depend on cellular immunological interactions in the periphery with their capacity to recruit and activate myeloid cells and T cells in the CNS [23].

B cells can cross BBB to lymph node–like follicles in the meninges, close to demyelinating lesions, and become CNS residents’ immune cells [64]; moreover, accumulation of B cells has been observed in the brain and spinal cordin EAE models [65]. In CSF of active MS patients, the B cell chemoattractant, CXCL13, is highly increased [66], and accumulation of CD80 and CD86 expressing inflammatory memory B cell has been observed [67]; it may be due to the production of some soluble factors via inflammatory astrocytes that promote CD86 expression on B cells [68].

The role of B cells as APCs and T cells activator may be a key in MS pathogenesis; some studies demonstrated that mice with B cells that lacked MHC-II failed to present antigen to T cells, and did not develop EAE when stimulated with recombinant human myelin oligodendrocyte glycoprotein (rhMOG), with diminished Th1 and Th17 T cell responses [69].

Depletion of B cells caused significant inhibition of the peptide encompassing the extracellular domains of myelin proteolipid protein (PLPECD) ability to induce EAE with inhibiting CD4^+^ T cell proliferation, activation, and secretion of pro-inflammatory cytokine [70]. According to Wu et al., treating RRMS with teriflunomide reduces MS pathogenicity due to expression downregulation of B cell CD80 and CD86 [71]. B cells can contribute in MS pathogenesis through its pro-inflammatory mediator role as B cells can cause changes in the cytokine secretion pattern, increase in pro-inflammatory cytokines such as IL-6 [53], TNF-α, lymphotoxin-α (LT), and GM-CSF [54, 72], and a decrease in regulatory cytokines like IL-10, IL-35, and transforming growth factor-β (TGFβ) [73–75]. GM-CSF production from B cells can be enhanced by STAT5 and STAT6. B cells produce less IL-10 and more GM-CSF in MS patients compared to healthy subjects. GM-CSF enhances IL-12 and IL-6 secretions from myeloid cells and aids in the development of Th1 and Th17 cells [54]. Also, IL-21 secreted by pathogenic Th17 cells might promote memory B cells survival and proliferation [55]. Therefore, memory B cells in MS have the ability to induce the activation of T cells and exhibit enhanced pro-inflammatory properties [55]. Mature naive B cells in MS showed abnormal proliferation and activation [56]. Interestingly, CD137^+^ B cells, that produce IL-6 production extensively with CD137L stimulation, have been investigated to accumulate in meningeal infiltrates from MS patients [76]. Anti-CD20 therapies decrease T cells count in the CSF and blood by 50% and 20%, respectively, and reduced the capacity of the remaining T cells to produce IFN-γ and IL-17 also [77, 78]. A recently discovered non-conventional pathway of B cells implication in MS is via secreting pathogenic microvesicles or exosomes that promote cultured oligodendrocytes and neuron death [79].

**Implication of Regulatory B cells and Neuroprotective Effect** Regulatory B cells (Bregs) are a subset of B cells population that acts to put down and control immune reactions. Bregs have the ability to produce IL-10 [80], TGFβ1 [80], and IL-35 [81] in MS and EAE. IL-10 + gut-derived IgA + plasma cells are a population of B cells that are observed to clearly enter the CNS in MS and EAE [82].

In vitro, B cells isolated from MS patients exhibit deficient IL-10 production than in B cells isolated from healthy controls [83]. In MS patients, IL-10-producing Breg cells are reduced during relapses compared with in remission [84], while other researchers cannot confirm the alteration of these cells frequency in MS [85]. Further research is required to validate this assumption.

Indeed, despite that, not all B cell–targeted treatments have a good outcome in MS treatment [86]. As mentioned about anti-CD20 therapies that cause selective loss of B cells express CD20, CD20^+^ B cells expressed on B cell lineages from pre-B cell to memory B cell, but not on pro B cell or plasma blast and plasma cells [87]. Atacicept, another B cell targeting drug, causes elimination of specific B cells subsets (plasma blasts and plasma cells) without affecting memory B cells [88]. This resulted in an increased pro-​inflammatory B cells activity and exacerbated CNS inflammation due to memory B cell activation enhancement that ends with disease exacerbation [88]. These results have explored the complicated B cells role in MS pathogenesis and further research is required for a better explanation of their exact role.

#### Role of Innate Immunity in MS

Innate immunity is believed to have a crucial role in MS initiation and progression by influencing the B and T cells to activate innate immune cells to express cytokines and other markers [89, 90].

Dendritic cells (DCs) are “professional antigen-presenting cells” that stimulate natural killer (NK) cell–mediated cytotoxicity [91] and promote naïve T cell differentiation and activation into either regulatory T cells (natural Tregs and induced Tr1 cells) or effector T cells (Th1, Th2, and Th17 cells) [92]. DCs also secrete osteopontin, a glycoprotein that participates in immune cells activation, differentiation, and chemotaxis. Indeed, increased osteopontin expression in brain lesions has been reported during EAE [93].

Phagocytes in the CNS can be descended from peripheral monocytes, which are macrophages, or from local microglial activation and are frequently difficult to distinguish from blood-borne phagocytes in tissue sections. Microglial cells, the most prevalent immune cells in the CNS, are considered CNS-resident macrophages with different activities such as the production of cytokines, phagocytosis, and antigen presentation [94]. It has a critical role in acute inflammatory response initiation and clearance of damaged tissues [89]. Infiltrating immune cells and resident glial cells have a role in neurodegeneration and proved to contribute to MS progression [95]. Astrocytes encourage recruitment of monocytes to the CNS; these monocytes are correlated with EAE severity [95].

Microglial cells also express TLRs (TLR 1–9) that make them essential for neuroimmune reactions generation [96]. Additionally, activated microglial participates in EAE worsening via increasing IL-6 production, macrophage inflammatory proteins, neurotropic factors, nitric oxide, and adhesion molecules [97]. Moreover, TNF-like weak inducer of apoptosis (TWEAK)–expressing microglial cells are responsible for severe myelin loss, neuronal injury, and vascular abnormalities in cortical lesions [98].

Natural killer (NK) cells “bridge” the gap between adaptive and innate immunity; CD56^dim^ and CD56^bright^ are the major NK cell subsets in humans. While the CD56^bright^ NK cell fraction produces anti-inflammatory cytokines that have “regulatory” activity, the CD56^dim^ NK cell subset mainly performs cytotoxic functions [99]. Till now, the exact role of NK cells in CNS autoimmunity is not clear. Further research is needed to explore how they contribute to immune regulation and inflammation [100].

#### Effect of Gut Microbiota and Its Derived Metabolites on CNS

**Fig. 2:**
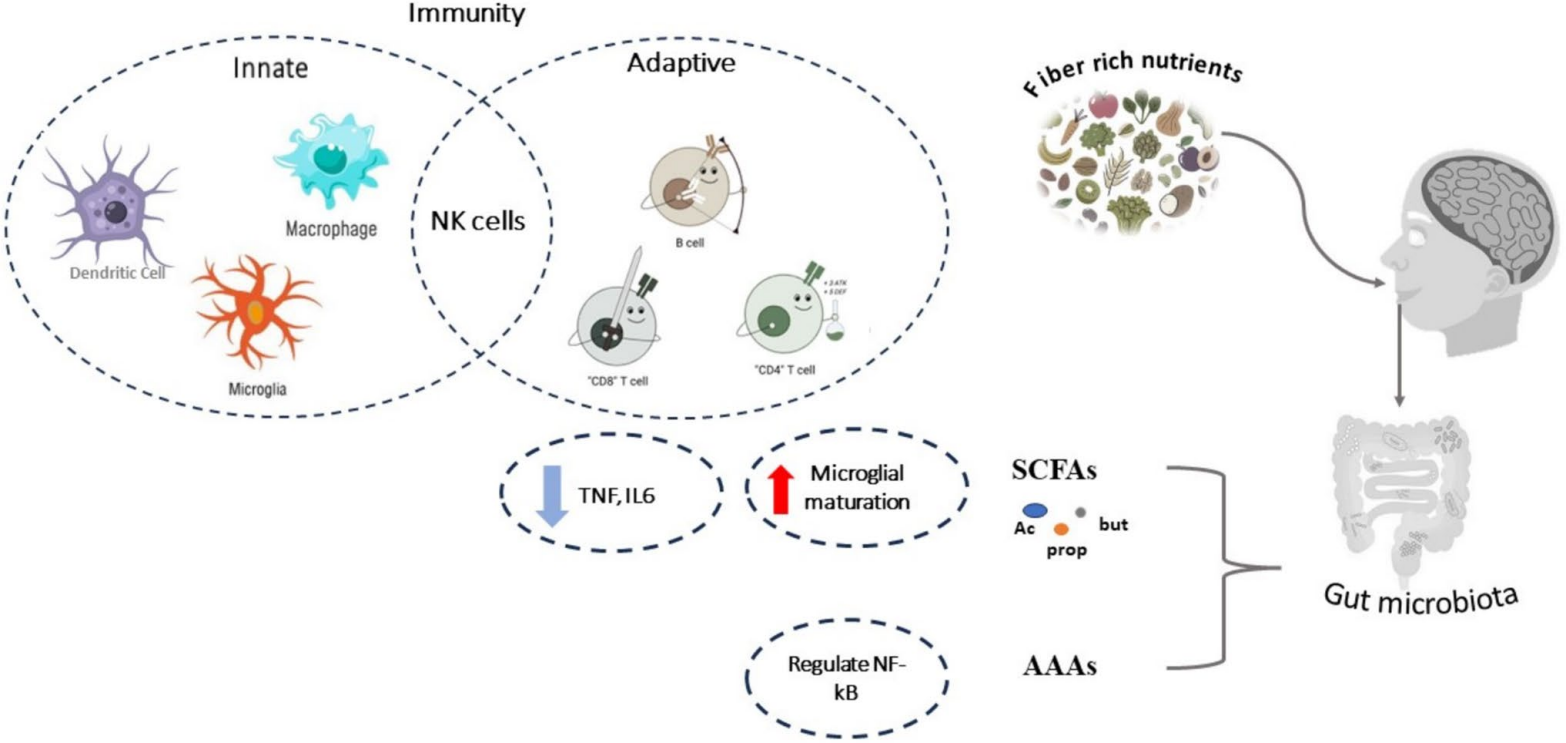
Schematic presentation for interaction between gut metabolites with immune system components. Short-chain fatty acids (SCFAs)—Ac, acetate; But, buterate; prop, propionate—activate innate immunity by increasing microglial cell maturation and decreasing tumor necrosis factor (TNF) and interleukins secretion while aromatic amino acid (AAAs) regulates NF-κB pathways

There are over 100 trillion microbes in the gastrointestinal tract (GIT), mostly in the colon [101]. This mixture of microbial communities, known as the GIT or GUT microbiota, consists of bacteria, fungi, eukaryotes, archaea, and viruses [7, 102]. Gut microbiota represents one of the environmental factors affecting health and any alteration in their composition is either to protect or increase susceptibility to chronic diseases [103]. Recently, it was observed that the gut microbiome can affect the CNS functions through bidirectional communication [104, 105]. Gut metabolites are defined as the end products or intermediates of the metabolism of its microbiota. These metabolites can reach the blood circulation, and some can pass blood–brain barrier (BBB) and affect CNS pathways and regulatory functions [102]. These metabolites have a role in neuroinflammatory, neuropsychiatric disorders, and neurodegenerative such as Parkinson’s disease (PD), autism spectrum disorders (ASD), Alzheimer’s disease (AD), and also multiple sclerosis (MS); their ability to ameliorate or aggravate some neuronal disorders has been reported as shown in Fig. 2 [106–109]. Dysbiosis means changes in gut microbiota balance; for example, the enrichment of specific pathogenic bacteria over the beneficial bacteria leading to release of some dangerous metabolites with a pro-inflammatory effects that compromise healthy gut barrier [110], and development of systemic inflammation via interaction with immune cells and modulates its behavior such as T cells, B cells, dendritic cells (DCs), and macrophages [111]. Additionally, gut dysbiosis has been associated with MS [112].

Gut microbiota produces three main metabolites that are associated with CNS health, short-chain fatty acids (SCFAs), aromatic amino acids (AAA), and trimethylamine N-oxide (TMAO).

**Firstly**, SCFAs such as butyrate, acetate, and propionate are produced from the fermentation of non-digestible carbohydrates such as starch from oats, beans, and legumes [113, 114]. Supplementation with SCFAs can alter several brain functions, inhibiting neuroinflammation via reducing the production of pro-inflammatory cytokines like TNF-α and IL-1β, and reducing IL-6 mRNA levels [115]. Moreover, SCFAs are ligands for free fatty acid receptors 2 and 3 (FFA2/FFA3) on several immune cells, and can modulate several pro-inflammatory cytokines secretion that induce neuroinflammation [116].

In germ-free (GF) mice, the morphological and genetic phenotypes of immature microglia were recovered partially through supplementation of the three major SCFAs (butyric acid, acetic acid, and propionic acid) [117]. Supplementing propionate to therapy naïve MS patients significantly increases Tregs and a decrease in Th1 and Th17 cells; also, it can reduce the annual relapse rate in those patients [118]. In addition, SCFAs play a role in ameliorating sensorimotor and communicative defects, decreasing anxiety-like behavior, reducing oxidative stress, regulating BBB, and improving memory loss associated with neuroinflammatory disorders [119]. In MS, both total SCFA production and the SCFA profile are altered and characterized by decreases in either acetate, butyrate, or propionate [120].

**The second group** is AAA which includes tryptophan, phenylalanine, and tyrosine; they have a crucial role in the microbiome gut-brain axis (MGBA) and also act as precursors to many secondary metabolites that work as neurotransmitters and affect the brain health [121]. For instance, tryptophan is the precursor of serotonin or 5-hydroxytryptamine (5-HT), indole, vitamin B3, and redox co-factors such as NAD(P)^+^ [122], while phenylalanine is the precursor of dopamine, norepinephrine, and epinephrine [123].

Studies reported that kynurenine, a tryptophan derivative, can decrease the activity of dendritic cells (DCs) and the natural killer (NK) in the CNS; however, its concentration in the brain leads to schizophrenia as well as depression [124]. Feeding mice with tryptophan-rich diet results in slowing brain aging through the regulation of NF-κB pathways and AMP-activated protein kinase (AMPK), thereby reducing inflammation and oxidative stress [125]. Phenylalanine produces amino acids such as aspartate, glutamate, and glycine that affect neurotransmission [126]. Aspartate and glutamate play as excitatory neurotransmitters, while glycine is an inhibitory neurotransmitter [127].

**Finally**, TMAO is a third gut metabolite that affects CNS health; they are produced from the fermentation of dietary constituents such as L-choline-rich food like meat, poultry, fish, dairy products, and eggs that may be a cause for dementia as a result of CD68 expression induction, which is dementia-associated marker [128]. It results in neuronal aging, increasing oxidative stress, and disrupting mitochondrial functions [127]. A study observed that increasing TMAO concentrations was correlated with increased astrocyte activation and pro-inflammatory cytokines [129].

Collectively, there is a two-way interaction of gut microbiota with CNS disorders and there is a brain-gut communication [117]. **Firstly**, studies on GF mice or mice treated with antibiotics demonstrate how specific microbiota can affect CNS physiology and neurochemistry; they showed increased numbers of immature microglia in different brain areas such as the cortex, hippocampus, and cerebellum in GF mice than specific pathogen-free (SPF) mice [130]. Their microglia have a decreased expression of genes that are responsible for maturation to an active phenotype, highlighting their immaturity and attenuation of several genes’ relevant pro-inflammatory cytokines, interferon responses, and effector processes [131]. In addition, the 3 major SCFAs, butyrate, acetate, and propionate, oral supplementation helped in inducing microglia maturation [132, 133]. **Secondly**, GF mice have reduced lymphoid follicles and lower Peyer’s patches number in the gut-associated lymphoid tissues (GALT); these tissues became smaller and have a lower T cells number than controls [134]. GF mice show a reduced susceptibility to disease in models of spontaneous [130] and actively induced EAE [135], rheumatoid arthritis (RA) [136], and inflammatory bowel disease [137].

#### Suggested Mechanisms of MS Pathogenesis Due to Impact of Gut Microbiota

In a healthy condition, intestinal epithelial cells (IECs) border the gut and use tight junctional proteins to keep bacteria and their byproducts apart from host cells and tissues. Nevertheless, dysbiosis may encourage a series of actions, such as the production of harmful toxins and the enrichment of pathogenic bacteria, which could result in a damaged gut barrier and a pro-inflammatory milieu production [138]. Pathogen-associated molecular patterns (PAMPs), such as LPS in gram-negative bacteria and other bacterial metabolites, play a significant role in systemic inflammation due to the increased gut permeability (leaky gut) [139]. We can summarize the potential pathogenic mechanism of gut microbiota as follows:

##### Chronic Inflammation

Proteobacteria, which raise oxidative stress and cause the generation of pro-inflammatory cytokines, are the source of the majority of pro-inflammatory stimulants such as LPS. The complex interacts with CD14 when LPS binds to the LPS binding protein, an acute phase protein. At the cell surface, this combination of CD14 and LPS-LPS binding protein interacts with TLR4/Myeloid differentiation factor 2 (MD-2), which in turn activates the cell via the NF-kB signaling pathway and causes the release of pro-inflammatory cytokines such IFN-y, TNF-α, IL-1β, and IL-8 that directly damage the epithelial barrier locally [140]. It has been demonstrated that LPS derived from gut bacteria, particularly *Bacteroides vulgatus*, induces pro-inflammatory endotoxin tolerance by binding to the MD-2/TLR4 receptor complex in intestine lamina propria CD11c + cells [141]. All things considered, gut dysbiosis can change the makeup of microorganisms, which can have pro- or anti-inflammatory immunological effects depending on variations in LPS immunogenicity. This can impact the aggravation or prevention of MS/EAE.

##### Increased Intestinal Permeability (Leaky Gut)

Because gut dysbiosis alters the makeup of the gut microbiota, it can change the homeostasis at mucosal surfaces. The generation of harmful metabolites and pro-inflammatory cytokines may rise, leading to the breakdown of the intestinal epithelial barrier with helpful compounds such as short-chain fatty acids (SCFAs) and other anti-inflammatory factors generated by gut microbiota are reduced [142]. Increased intestinal permeability may also help the gut microbiota activate peripheral immune cells, particularly gut-associated lymphoid tissue (GALT), and transfer toxic compounds into the bloodstream. Gut-specific inflammation, which can be a symptom of neurological demylination conditions such as schizophrenia and Crohn’s, is a predisposing characteristic of leaky gut [143]. In addition to gut dysbiosis, MS patients have elevated gut permeability and high levels of pro-inflammatory cytokines in their serum, such as IL-1β, TNF-α, and IL-6 to correlate with leaky gut [144].

##### Macrobiotic Induction of Pro-inflammatory T cell

Self-reactive, myelin-specific CD4 + T helper cells are believed to cause MS, with Th17 cells being the most implicated lineage. Th17 cells are characterized by their production of the pro-inflammatory cytokine IL-17 and migrate to the CNS during active disease [145]. Therefore, deregulation of Th17 cell growth and differentiation, mediated by gut microbiota, is thought to have a role in the development or progression of MS. *Acinetobacter calcoaceticus* and *Akkermansia muciniphila* are two bacterial species that have been linked to MS because they can stimulate Th17 induction and pro-inflammatory activities [146]. Colonization by *Akkermansia muciniphila* has also made EAE worse in vivo. These immunostimulatory bacteria can either directly or indirectly trigger Th17 cell responses by producing metabolites. By stimulating the aryl hydrocarbon receptor (AhR), *Lactobacillus reuteri* metabolize tryptophan to boost CNS autoimmunity and IL-17 production [147].

##### Pro-inflammatory B cells Induction by Microbiota

More than 90% of MS patients exhibit positive immunoglobulin G (IgG) oligoclonal bands in their cerebrospinal fluid (CSF), indicating abnormalities in the amount and quality of immunoglobulins in the CSF due to infiltration of B cells [148]. In EBV or MS, the gut microbiota’s impact on pathogenic B cell responses is controversial; the effectiveness of B cell depletion treatments in MS and the strong evidence that the development of clinical symptoms is associated with Epstein-Barr virus (EBV) infection and anti-EBNA antibody levels highlight the importance of these abnormalities [149]. On the other side, B cells’ anti-inflammatory, gut microbiota–dependent function in multiple sclerosis has been clarified. IgA + plasma cells (PCs) are considerably diminished in the stomach during EAE, and crucially, the elimination of PCs and plasma blasts aggravated EAE [82]. Therefore, IgA + B cells exhibit selectivity for MS-associated immunostimulatory bacterial strains and can pass the blood–brain barrier during active multiple sclerosis, according to a follow-up investigation. Nevertheless, these IgA + B cells do not cross-react with the self-antigen and were strong IL-10 producers [150].

##### Regulatory T cell Modulation by Microbiota

Multiple research on GF mice have proved for the first time the link between the immune system and the gut microbiota in immune disease development [135, 151]; GF mice exhibited weakened MS form with decreased pro-inflammatory cytokines and increased regulatory T cells (Treg) than normal colonized mice [151]. Early antibiotic administration in EAE rats disrupts the gut microbiota composition with reduced SCFAs levels, resulting in stronger immune response and EAE aggravation [152]. Offspring of mice inhabited with specific bacteria in GIT that mostly enhance the response of T helper 17 have a higher probability of suffering from neurodevelopmental diseases [153]. Other research groups observed also that changes in gut microbiota composition can influence the natural killer T (NK) cell populations [154].

In MS patients, gut microbiota is significantly changed in specific microbial taxa compared to healthy controls [155, 156] confirming gut microbiota role in MS development. The 16S ribosomal RNA sequencing of microbiota of MS patients in the relapse phase showed a decrease in phylum *Bacteroidetes* such as *Bacteroides* and *Parabacteroides* species and an increase in phylum *Firmicutes* such as *Dorea* and *Blautia* species if compared to healthy subjects or MS patients in remission period [146, 157]. Moreover, there was lower *Prevotella*, *Bacteroidetes* phylum, that produces the anti-inflammatory propionate [155, 156] and higher *Streptococcus mitis* (*S. mitis*) and *Streptococcusoralis* (*S. oralis*), *Firmicutes* phylum, that promote Th17 cells differentiation [157] in MS patients compared to control. Relapsing–remitting multiple sclerosis (RRMS) patients showed a lower prevalence of both *Prevotella* [158, 159] and *Clostridium* which is linked to Th17 cell expansion and enhances the production of IL-10 (the anti-inflammatory cytokine) and Treg cells in peripheral compartments respectively [160, 161]. The presence of another gut microbiota residents a short filamentous bacteria called symbiont *Bacteroides fragilis* (SBF) which promote neurological inflammation through its metabolites via macrophages activation, that contribute to IL 23 synthesis, also play as APCs that induce differentiation of T cells into Th17 cells [162]. Taken together, these investigations reveal that the gut microbiota contributes directly to the pathological process of MS by controlling Th17 proliferation at the gut level [163].

Interestingly, gut commensal can induce Tregs in the gut [164]; the most prevalent capsular polysaccharide produced by *B. fragilis*, polysaccharide A (PSA), drives the transformation of CD4^+^ T cells to Foxp3 + Tregs which release IL-10 and suppress Th17 responses by activating Toll-like receptor 2 (TLR-2) and protects against MS [165]. Additionally, oral treatment of *B. fragilis* PSA has been linked to increased frequencies of CD39^+^ Tregs in the lymph nodes of CNS [166] and a lower MS “clinical” score in an IL-10- and TLR2-dependent manner [167].

Further examples for the pathogenic microbiota and their role on the immune components affecting the MS pathogenesis can be summarized in Table 1.

**Table 1:** The effect of immune components and responsible microbiota on MS pathogenesis

Immune system	Types of cells	Implication in MS pathogenesis	Responsible microbiota	Reference
A- Adaptive immunity	Effector CD4^+^ T cells	Th1 cells produce IL2, IFN-γ, and TNF-α	• *Clostridium* species • *Bacteroides*, *Lactobacillus*, and *Streptococcus* genera	• Legroux and Arbour (2015) • Koji et al. (2013) • Geuking et al. (2011)
		• Th2 cells produce pro-inflammatory IL-4, IL-5, and IL-13	• *Parabacteroides distasonis* • *Akkermansia muciniphila*	• Wang et al. (2020) • Eagle et al. (2017)
		• Th17 cells produce IL-17	• Segmented filamentous bacteria • *Escherichia coli*, *Bifidobacterium adolescentis*, *Staphylococcus aureus*, and *Candida albicans*	• Wang et al. (2020) • Magarian et al. (2017)
	Effector CD8^+^ T cells	• Produce IL-17 and IFN-γ; cytotoxic function that causes axonal damage	• Lactic acid bacteria including *Pediococcus acidilactici*	• Larochelle et al. (2015a), Melzer et al. (2009)
	B cells	• Antigen-presenting cells for T cells	• Segmented bacteria	• Schirmer et al. (2014)
		• Production of CSF-​restricted IgG OCBs • IgA binding	• *Eggerthella lenta* and *Bifidobacterium adolescentis*	• Schirmer et al. (2014)
		• Production of pro-inflammatory cytokines GM-CSF, TNF-α, IL-6, and LT-α	• *Bacteroidetes* and *Proteobacteria* phyla	• Li et al. (2015a), Bar-Or et al. (2010), Li et al. (2017, 2016)
		• Production of inflammatory cytokines: transforming growth factor β1, IL-35, and IL-10	• *Bacteroides fragilis*	• Barr et al. (2012), Bar-Or et al. (2018) • June et al. (2011)
B- Innate immunity	Dendritic cells	• T cells activation to effector T cells	• *Candida kefyr*	• Gilliet and Liu (2002)
		• NK cell–mediated cytotoxicity induction	• *Bifidobacterium bifidus*	• Fernandez et al. (1999)
		• Production of IL-6, IFN-γ, TNF-α	• *Bacteroidetes* and *Proteobacteria* phyla	• Huang et al. (1999)
		• IL-23 and osteopontin	*Bifidobacterium bifidum*	• Vaknin-Dembinsky et al. (2008) • Zhang et al. (2024)
	Microglial cells/macrophages	• Production of IL-6, IL-17, macrophage inflammatory proteins, neurotropic factors, nitric oxide, adhesion molecules, and TWEAK • Expression of myeloperoxidases and ROS	• *Actinobacteria*	• Kawanokuchi et al. (2008), Serafini et al. (2008) • Gray et al. (2008), Raivich and Banati (2004)
	NK cells	• Cytotoxic activity toward oligodendrocytes • Production IFN-γ and TNF-α.	• *Bifidobacterium*	• Lünemann et al. (2008) • Schleinitz et al. (2010)

#### Potential Future Therapeutic Roles of Microbiome in MS

Targeted therapies that alter the gut microbiota to have a “healthier” makeup are still a promising treatment option. Numerous strategies, such as nutrition, probiotics, synbiotics, and fecal microbiome transplantation (FMT), are employed to alter gut microbiota and have promise in MS. Supplementing food with *Prevotella histicola* was demonstrated to be just as effective as COPAXONE® in reducing inflammation and demyelination in the brain and improving EAE [168]. To encourage the growth of good gut flora, a combination of probiotic supplements and dietary changes—known as synbiotic therapy—may also be crucial [169]. This strategy has showed potential in animal models of multiple sclerosis, where supplementing with *Parabacteroides distasonis* and *Aldercrutzia equolifaciens* together with a diet high in isoflavones reduced EAE. Another intriguing treatment option with promise for GI disorders like *Clostridium difficile* infection is fecal microbial transplant (FMT) [170]. It is still difficult to define what makes up a “healthy” microbiome that has the capacity to reduce inflammation and multiple sclerosis.

## Conclusion

This review discussed the significant role of the immune system through its two arms: the adaptive and innate immunity in MS pathology that ends with myelin sheath degeneration and axonal loss. Moreover, we discussed the insight of the gut microbiota composition and the impact of its metabolites on immune responses and MS severity. There are some microbiota members associated with MS exacerbation through enhancement of effector T cell proliferation and inflammatory cytokines production. Genera like *Dorea*, *Blautia*, *Streptococcus*, *Akkermansia*, *Pedobacteria*, *Methanobrevibacter*, *Flavobacterium*, and *Proteobacteria* are higher in patients of MS than in healthy subjects. Conversely, others have a beneficial effect by enhancing proliferation of Treg cells and aiding in relieving MS symptoms.

## Supplementary Information

Below is the link to the electronic supplementary material.

ESM 1 (DOCX 33.6 KB)

## Data Availability

No datasets were generated or analyzed during the current study.
