# Supplementary material for: Multiple Sclerosis: A Story of the Interaction Between Gut Microbiome and Components of the Immune System
Source: Mol Neurobiol. 2025 Feb 11;62(6):7762–75. doi: 10.1007/s12035-025-04728-5 (PMC12078361; doi:10.1007/s12035-025-04728-5)
Supplement: Supplementary file 1 — (DOCX 33.6 KB) [file 12035_2025_4728_MOESM1_ESM.docx]

**List of tables:**

**S1: The effect of immune components in MS pathogenesis**

| Immune system | Types of cells | Implication in MS pathogenesis | Reference |
| --- | --- | --- | --- |
| A- Adaptive Immunity | Effector CD4^+^ T Cells | - Th1 cells produce IL2, IFN-γ and TNF-α | - Legroux and Arbour, 2015). |
|  |  | - Th2 cells produce proinflammatory IL-4, IL-5 and IL-13 | - (Wang et al., 2020). |
|  |  | - Th17 cells produce IL-17 | - (Wang et al., 2020). |
|  | Effector CD8^+^ T Cells | - Produce IL-17 and IFN-γ that; cytotoxic function that causing axonal damage. | - (Larochelle et al., 2015a), (Melzer et al., 2009). |
|  | B Cells | - Production of CSF-​restricted IgG OCBs. | - (Schirmer et al., 2014) |
|  |  | - IL-6. | - Barr et al., 2012) |
|  |  | - GM-CSF, TNF-α, and LT-α and T cells activation. | - (Li et al., 2015a), (Bar-Or et al., 2010; Li et al., 2017, 2016). |
| B- Innate Immunity | Dendritic Cells | - T cells activation to effector T cells | - Gilliet and Liu, 2002) |
|  |  | - NK cell-mediated cytotoxicity induction | - (Fernandez et al., 1999), |
|  |  | - Production of IL-6, IFN-γ, TNF-α | - (Huang et al., 1999), |
|  |  | - IL-23 and osteopontin (Hur et al., 2007). | - (Vaknin-Dembinsky et al., 2008 |
|  | Microglial Cells/ Macrophages | - Production of IL-6, IL-17, macrophage inflammatory proteins, neurotropic factors, nitric oxide, adhesion molecules and TWEAK - Expression of myeloperoxidases and ROS | - (Kawanokuchi et al.2008), (Serafini et al., 2008). - (Gray et al., 2008), (Raivich and Banati, 2004). |
|  | NK cells | - Cytotoxic activity toward oligodendrocytes. - Production IFN-γ and TNF-α.. | - (Lünemann et al., 2008). - (Schleinitz et al., 2010). |
